# p53R2 as a novel prognostic biomarker in nasopharyngeal carcinoma

**DOI:** 10.1186/s12885-017-3858-4

**Published:** 2017-12-13

**Authors:** Jiewei Chen, Shuman Li, Yongbo Xiao, Xuan Zou, Xinke Zhang, Mingshu Zhu, Muyan Cai, Dan Xie

**Affiliations:** 1 Sun Yat-sen University Cancer Center ; State Key Laboratory of Oncology in South China; Collaborative Innovation Center for Cancer Medicine, Guangzhou, China; 2 Department of Pathology, Sun Yat-sen University Cancer Center, No. 651, Dongfeng East Road, Guangzhou, 510060 China; 3 Department of Pathology, The Sixth Affiliated Hospital, Sun Yat-sen University, Guangzhou, 510655 China

**Keywords:** Nasopharyngeal carcinoma, p53R2, Immunohistochemistry, Prognosis

## Abstract

**Background:**

p53R2 is a target of p53 gene, which is essential for DNA repair, mitochondrial DNA synthesis, protection against oxidative stress, chromosomal instability, chronic inflammation and tumorigenesis. This study is aimed to investigate the expression of ribonucleotide reductase (RR) subunit p53R2 in nasopharyngeal carcinoma and its significance in the prognosis.

**Methods:**

The expression levels of p53R2 in 201 patients with NPC were examined by immunohistochemical assay. The correlations of p53R2 expression and clinicopathological features of nasopharyngeal carcinoma patient were analysed by chi-square test. The Kaplan-Meier survival analysis and Cox multivariate regression model were used to analyze the prognostic significance of the patients with NPC.

**Results:**

Immunohistochemical results showed that p53R2 was positively expressed in 92.5% (186/201) of nasopharyngeal carcinoma and the high expression rate was 38.3% (77/201). Further analysis observed that the negative correlation between expression of p53R2 and pT status had statistical significance (*P* < 0.05). Kaplan-Meier survival analysis found that the mean survival time of patients with high expression of p53R2 was 143.32 months, while the patients with low expression level of p53R2 was 121.63 months (*P* < 0.05). Cox regression analysis suggested that p53R2 protein expression could be used as an independent prognostic factor for nasopharyngeal carcinoma (*P* < 0.05).

**Conclusions:**

This study drew a conclusion that p53R2 could be used as a prognostic biomarker indicative of the favorable outcome for patients with nasopharyngeal carcinoma.

## Background

Nasopharyngeal carcinoma (NPC) is the malignant cancer occurring on the top and lateral wall of nasopharynx cavity [1], which is prevalent in southeast Asia especially in southern China. Most of the NPC patients are diagnosed at the stage of III or IV, and the 5 years survival rate is 50%–60% [2]. It has been proved that Epstein-Barr virus infection, genetic susceptibility, environmental factors, dysfunction of oncogenes or suppressor genes and life styles are all associated with NPC tumorigenesis [3]. The process of nasopharyngeal carcinoma from mucosal epithelium of the nasopharynx, to low-grade dysplastic epithelium, high-grade dysplastic epithelium, invasive and metastasis cancer involves in multiple genes alteration, for example, alleles loss on 3p, 9p, 11q, 13q, 14q, 16q and gained on chromosomes 8,12 [4, 5]. p53R2 located on chromosome 8q23.1 is a target of p53 gene. When DNA is damaged, the cell cycle is blocked at G1 and G2 stage. Subsequently, p53R2 is upregulated and accumulated in the nuclear to provide dNTP to repair the damaged DNA [6, 7]. Different phenotypes of p53R2 have been found in various human cancers. In small cell lung cancer and esophageal cancer, high level of p53R2 expression has been shown to be a biomarker of tumor invasion and worse prognosis, which indicates p53R2 may be an oncogenic role in these cancers [8, 9]. While in colorectal cancer, overexpression of p53R2 indicates a good outcome for the patients, suggesting that p53R2 may be a tumor suppressor [10, 11]. However, it’s still unclear what’s the expression status of p53R2 expression in NPC and its clinicopathological significance. Here we used IHC to evaluate the protein level of p53R2 in nasopharyngeal carcinoma tissues and apply the statistic analysis methods to identify the association between p53R2 and the prognostic significance of nasopharyngeal carcinoma.

## Methods

### Patients and specimens

In this study, 201 specimens of NPC in Sun Yat-sen University Cancer Center from January 2001 to October 2012 were collected. The cases selected were based on the following criteria: pathologically confirmed as nasopharyngeal cancer with available biopsy specimens for immunohistochemistry; no previous malignant disease or a second primary tumor; without radiotherapy, chemotherapy and surgery treatment history; completed follow-up data. Patients who had no complete clinical follow-up data or had died from other unknown reasons were excluded. The pTMN stage was defined based on the sixth edition TNM classification criteria established by the International Union Against Cancer (UICC, 2002). All the experiments done in this study were approved by the institute research medical ethics committee of Sun Yat-sen University.

### Immunohistochemistry (IHC)

IHC was performed using standard EnVision method. The paraffin-embedded tissue blocks were cut into 3 μm thick sequential sections, the slides were dried and deparaffinized in xylene, rehydrated through graded alcohol, immersed in 3% hydrogen peroxide for 10 min to block endogenous peroxidase activity and antigen retrieved by pressure cooking for 3 min in citrate buffer (pH = 6). Then the slides were incubated with 5% BSA for 15 min to reduce nonspecific reaction. Subsequently, the slides were incubated with the rabbit monoclonal antibody anti-p53R2 (Abcam, ab154194, 1:400 dilution) for 50 min at 37 °C. The slides were sequentially incubated with a secondary antibody (Envision, Dako, Denmark) for 30 min in the incubator at 37 °C, and stained with DAB (3,3-diaminobenzidine). Finally, the sections were counterstained with Mayer’s hematoxylin, dehydrated and mounted. A negative control was obtained by replacing the primary antibody with a normal rabbit IgG.

### IHC evaluation

p53R2 staining was mainly observed at the cytoplasm, and the positively stained cells were brown or yellow. Immune reactivity was scored by evaluating the number of positive cells and the positive intensity score: (i) The percentage of positive tumor cells: take 5 fields every slide to counter the percentage in 5% increments from 0 to 100% (0 indicates negative staining). (ii) Positive intensity score: negative (0), weak (1), moderate (2) and strong (3). (iii) The scores obtained from intensity and the proportion (0–300 scores). We used the ROC curve to determine the cut-off value of p53R2 expression level in NPC. Two pathologists who were blind to the information of patients performed the scoring. If the results were different, then a third pathologist would participate to confirm the score.

### Statistical analysis

SPSS software (version 21.0, SPSS, Chicago, IL) was used to operate the analysis. ROC analysis was performed to determine the cut-off value for p53R2 expression. We applied χ^2^ test to evaluate the relationship between p53R2 and NPC patients’ clinicopathological characteristics. Univariate analysis was performed by the Kaplan-Meier. Cox regression analysis was employed to identify the independent prognostic factor. Two–tailed *P* value less than 0.05 was considered statistically significant.

## Results

### p53R2 expression in NPC tissues examined by IHC

**Fig. 1:**
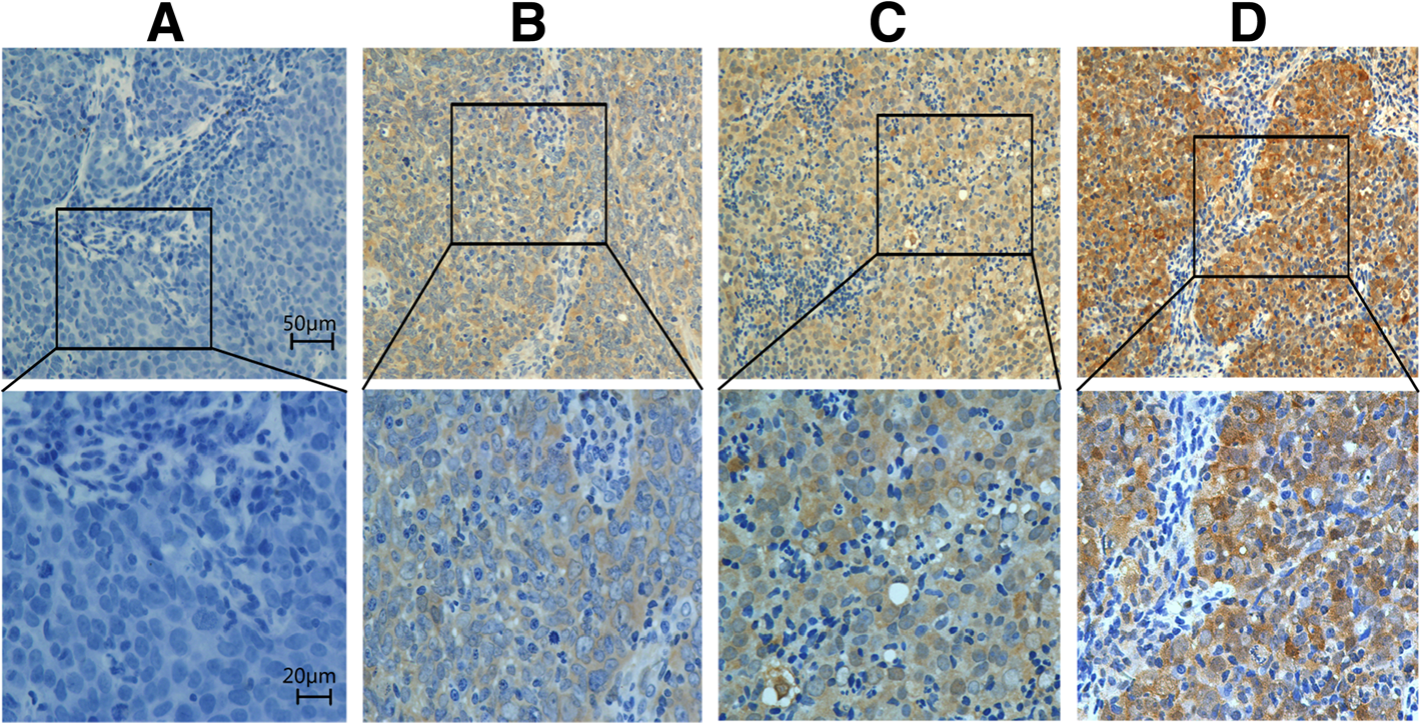
Expression of p53R2 protein in NPC tissues. **a**, Negative expression; **b**, Low expression; **c**, Moderate expression; **d**, Strong expression

The positive expression of p53R2 by IHC analysis in nasopharyngeal carcinoma was primarily a cytoplasm pattern (Fig. 1) and the positive expression rate was 92.5% (186/201).

### Cut-off value for p53R2 expression

**Fig. 2:**
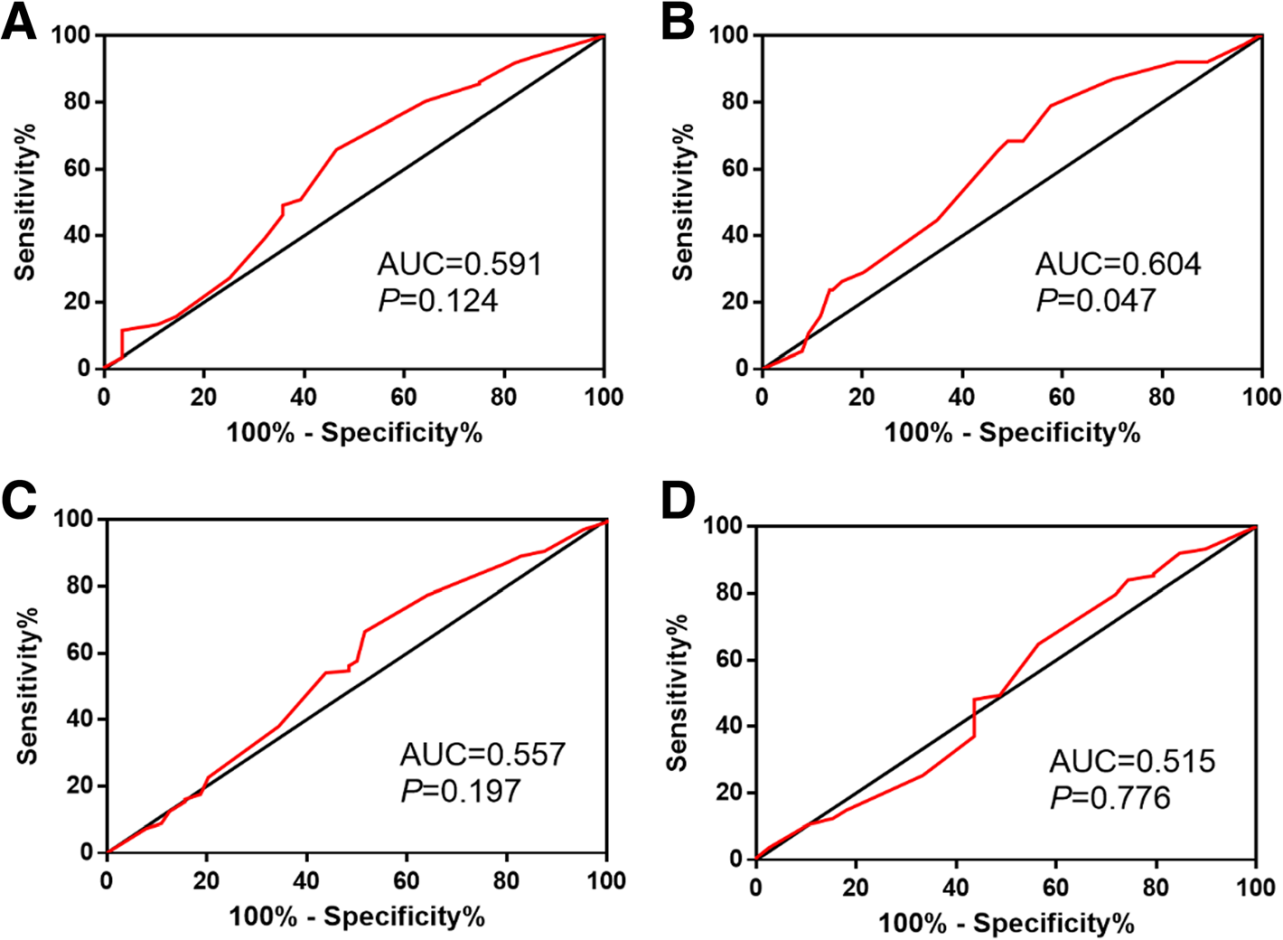
ROC curve analysis was employed to determine the cut-off value for high p53R2 expression in NPC. The sensitivity and specificity for each outcome were plotted: pN status (**a**), survival outcome (**b**), pT status (**c**), clinical stage (**d**)

ROC curve was used to identify the cut-off for p53R2. The point with both maximum sensitivity and specificity was chosen as the cut-off point [12]. Area under the curve (AUC) and *P* value were shown in Table 1. The sensitivity and specificity for each clinicopathological feature were plotted (Fig. 2). Therefore, we used the survival status as a state variable. The ROC curve analysis revealed that the cut-off value of the expression of p53R2 protein was 150 (*P* < 0.05).

**Table 1:** AUC operating characteristic curve for each clinicopathological feature

Feature	AUC (95% CI)	*P* value^a^
T stage	0.557 (0.468–0.645)	0.197
N stage	0.591 (0.469–0.712)	0.124
Survival status	0.604 (0.509–0.698)	0.047
Clinical stage	0.515 (0.405–0.624)	0.776

^a^Chi-square tests

### Association of p53R2 expression with NPC patients’ clinicopathological features

Further analysis showed that expression of p53R2 was significantly correlated with T stage (Table 2, *P* = 0.043) and there was no significant association between p53R2 expression and other clinicopathological features, such as patient sex, age, lymph node metastasis, clinical stage, therapeutic regimen, relapse (Table 2, *P* > 0.05). As shown in this table, high-level of p53R2 was observed in 48.4% of stage T1 + T2 patients and in 33.6% of stage T3 + T4 patients.

**Table 2:** Correlation between the p53R2 expression and clinicopathological variables in NPC patients

Variable	Expression of p53R2			
	All cases	Low	High	*P* value
Gender				0.217
Female	57	39 (68.4%)	18 (31.6%)	
Male	144	85 (59.0%)	59 (41.0%)	
Age				0.593
≤45.89^a^	104	66 (63.5%)	38 (36.5%)	
>45.89	97	58 (59.8%)	39 (40.2%)	
Clinical stage				0.450
I-II	39	22(56.4%)	17 (43.6%)	
III-IV	162	102(63.0%)	60 (37.0%)	
T stage				0.043
T1 + T2	64	33 (51.6%)	31 (48.4%)	
T3 + T4	137	91 (66.4%)	46 (33.6%)	
N stage				0.469
N0	28	19 (67.9%)	9 (32.1%)	
N1 + N2 + N3	173	105 (60.7%)	68 (39.3%)	
Therapy^b^				0.205
Regimen 1	50	34 (68.0%)	16 (32.0%)	
Regimen 2	72	46 (63.9%)	26 (36.1%)	
Regimen 3	31	14 (45.2%)	17 (54.8%)	
Regimen 4	48	30 (62.5%)	18 (37.5%)	
Relapse				0.543
Yes	30	20 (66.7%)	10 (33.3%)	
No	171	104 (60.8%)	67 (39.2%)	

^a^mean age; ^b^Regimen 1, radiation therapy;Regimen 2, chemoradiotherapy; Regimen 3, induction + radiation therapy; Regimen 4, induction + chemoradiotherapy

### The relationship between p53R2 expression status and clinicopathological characteristics and NPC patients’ survival

**Fig. 3:**
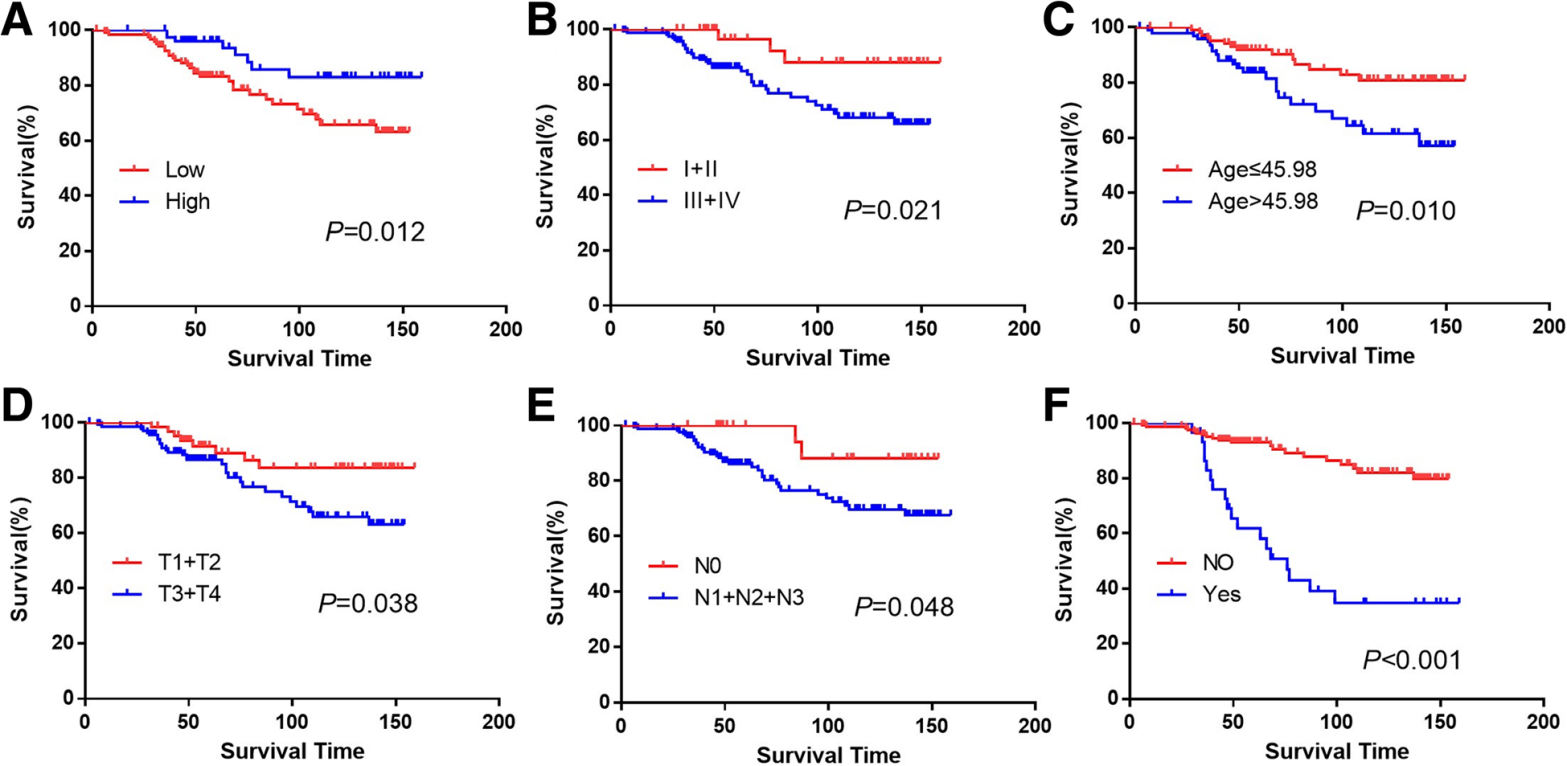
Different prognostic factors for survival outcome in 201 patients with NPC. The overall for each outcome were plotted: p53R2 expression (**a**), clinical stage (**b**), age (**c**), pT status (**d**), pN status (**e**), relapse (**f**)

In this present study, the survival analysis showed that patients with high p53R2 expression had a better survival (*P* = 0.012, Fig. 3a). To explore the prognostic factor for NPC, we calculated the influence of the clinicopathological features on the prognosis of NPC, the mean survival period of patients with clinical stage I-II (mean: 149.60 months) was longer than that with clinical stage III-IV (mean: 126.65 months) (*P* = 0.021, Fig. 3b). The mean overall survival period for patients with older age was shorter than that with younger age; the difference was statistically different (*P* = 0.010, Fig. 3c). There was also a better survival for patients with stage T1 and T2 compared to patients with T3 and T4 (*P* = 0.038, Fig. 3d). We also observed a significantly different survival rate between the patients with N0 and the patients with N1–3 (*P* = 0.048, Fig. 3e). Recurrence was also found to be a significant prognostic factor for NPC (*P* < 0.001, Fig. 3f). All the detailed data were shown in Table 3.

**Table 3:** Univariate survival analysis of different prognostic factors in 201 patients with NPC

Variable	All cases	Mean survival (months)	Chi-square value	*P*-value
Gender			2.291	0.130
Male	144	125.36		
Female	57	142.17		
Age			6.644	0.010
≤45.89	104	141.09		
>45.89	97	118.95		
Clinical stage			5.310	0.021
I-II	39	149.60		
III-IV	162	126.65		
T stage			4.323	0.038
T1 + T2	64	142.54		
T3 + T4	137	123.32		
N stage			3.915	0.048
N0	28	146.06		
N1 + N2 + N3	173	129.18		
Therap**y**			2.841	0.417
Regimen 1	50	122.82		
Regimen 2	72	133.64		
Regimen 3	31	127.82		
Regimen 4	48	122.49		
Relapse			32.95	0.000
No	171	138.15		
Yes	30	91.75		
Expression				
Low	124	121.63	6.347	0.012
High	77	143.32		

**Fig. 4:**
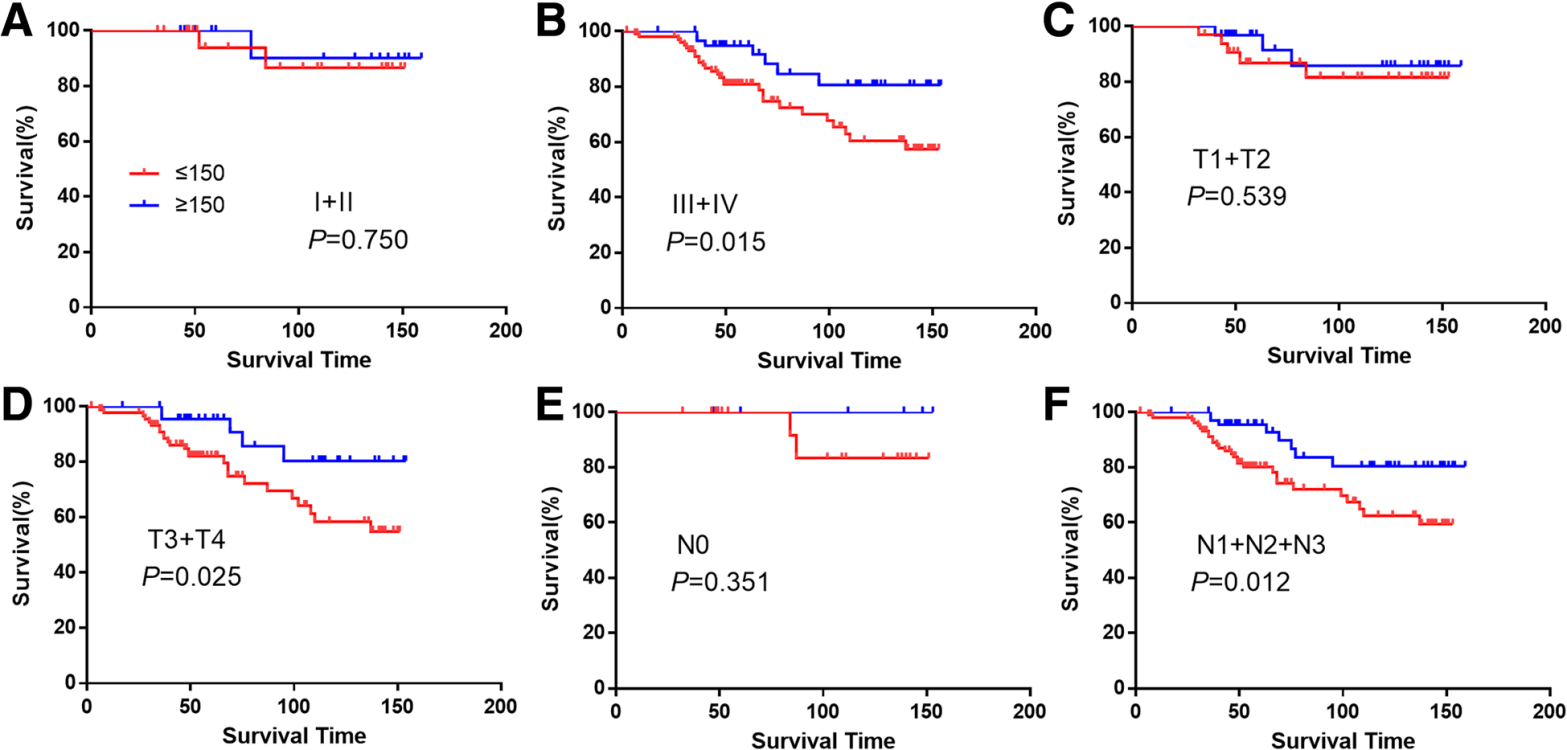
Kaplan-Meier survival analysis of p53R2 expression in subsets of NPC patients with different clinical stage and pT/pN stage. Clinical stage I + II (**a**), Clinical stage III + IV (**b**), pT1 + T2 (**c**), pT3 + T4 (**d**), pN0 (**e**), pN1 + N2 + N3 (**f**)

To investigate the impact of the p53R2 protein expression on the survival of NPC patients with different subgroups, further analysis was performed regarding p53R2 expression in subsets of NPC patients in different clinical stage, pT stage, pN stage. We observed that the expression of p53R2 was a prognostic factor for stage III + IV (*P* = 0.015), pT3 + T4 (*P* = 0.025), pN1 + N2 + N3 (*P* = 0.012, Fig. 4).

### Independent prognostic factors for NPC patients

The factors that had a significant difference in univariate analysis were further tested in the cox regression analysis, and the results suggested that p53R2 was an independent prognostic factor. Additionally, age and tumor relapse were independent prognostic factors for NPC patients as well (Table 4).

**Table 4:** Cox multivariate analysis of prognostic factors on overall survival

Variable	Hazards ratio	95% CI*	*P* value
Age (>45.89 vs. ≤45.89)	3.14	1.58–6.25	0.001
Clinical stage (III-IV vs. I-II)	3.27	1.00–10.67	0.050
Relapse (Yes vs. No)	5.97	3.10–11.49	0.000
p53R2 expression (High vs. Low)	0.39	0.18–0.85	0.018

*CI, confidence interval;

### The relationship between the expression of p53R2 and the overall survival rate

We utilized Kaplan-Meier analysis to evaluate the relationship between the expression of p53R2 and the survival rate of NPC patients (Table 3). In the high p53R2 expression group: this subgroup had a mean survival time of 143.32 months; the 5-year survival rate was 96.00%, and the 10-year survival rate was 82.90%. However, in low p53R2 expression group: the mean survival period was 121.63 months, the 5-year survival rate was 83.20%, and the 10-year survival rate was 63.20% (Table 5).

**Table 5:** The expression of p53R2 for five-year survival rate and ten-year survival rate

p53R2 expression	Mean survival time (months)	Five - year survival rate (%)	Ten - year survival rate (%)
Low	121.63	83.20	63.20
High	143.32	96.00	82.90

## Discussion

p53R2, a ribonucleotide reductase small subunit, belongs to the ribonucleotide reductase family. p53R2 offers dNTPs for DNA replication and repair [13]. p53R2 is essential for DNA repair, mitochondrial DNA synthesis, protection against oxidative stress, chromosomal instability, chronic inflammation and tumorigenesis [7, 14, 15]. Recent study has found that p53R2 point mutation in HCT116 (a colorectal cancer cell line) could lead to ribonucleotide reductase (RR) activity attenuation and dysfunction of DNA repair [16]. Another study also indicated that in nontransformed cells, p53R2 was critical for maintaining mtDNA and repairing UV damaged DNA during quiescence [17].

Herein, we estimated the protein status of p53R2 in 201 NPC specimens by IHC. The result demonstrated that p53R2 was positive in 92.5% of the NPC, and further analysis revealed a significant correlation between p53R2 expression and pT stage by chi-square test. Univariate Kaplan-Meier analysis indicated that the status of p53R2 expression have a significant impact on patient survival. Cox multivariate analysis found that p53R2 was an independent prognostic factor for NPC. Taken together, our results suggest that p53R2 expression is a reliable biomarker for prognosis of NPC.

There are a few reports about the relationship between p53R2 expression and the prognosis of human cancers. In a study on colorectal cancer, high-level of p53R2 expression indicated patients having a longer survival period and could be a favorable prognostic factor [10, 11]. In consistent with this study, our data show that p53R2 expression is negatively correlated with clinicopathological parameters and predicts a good outcome of NPC patients. Several previous studies reported that the positive staining of p53R2 examined by IHC, was observed dominantly in the cytoplasm of tumor cells, such as colon cancer, lung cancer and esophageal cancer [9, 18, 19]. In response to DNA damage stress, p53R2 will translocate from cytoplasm to nucleus [20]. M2B may translocate from the cytoplasm into the nucleus and allow dNTPs to initiate DNA synthesis in KB cells under physiological conditions [21].

More and more researchers show that p53R2 plays a key role in many biological processes and diseases including tumors. In the field of mitochondrial DNA disorder, RRM2B (encoding p53R2) is critical for mtDNA copy number and its dominant-negative or gain-of-function mutations is a major reason for mtDNA deletions and adPEO [22]. RRM2B-mousa had a renal dysgenesis and died of sever renal dysfunction at 14th week. So p53R2 is essential for the maintenance of normal renal function [23]. By contrast, there are some studies pointing out that p53R2 could promote the aggression of tumor [8, 9]. p53R2 enhanced the invasion of cancer through E-cadherin/β-catenin pathway [24]. In esophageal squamous cell cancer, p53R2 was significantly correlated with the infiltration depth, lymph node metastasis and poor prognosis [9]. p53R2 was also an adverse biomarker for the non-small cell lung cancer [8]. Moreover, p53R2 was further found to be correlated with lymph node metastasis, infiltration, general stage of the tumors in oral cancer and melanoma, while there is no relationship found between p53R2 and gastric cancer [25–27].

Our result showed that p53R2 was a protective factor for the prognosis of nasopharyngeal carcinoma. These results may suggest p53R2 has the ability to repair the damaged DNA and inhibit tumor invasion. The expression of p53R2 is regulated by p53 in response to genotoxic stimulation, such as UV and chemical therapy (i.e., adriamycin) [6, 28], and the depletion of p53R2 can enhance the DNA damage caused by adriamycin [29]. After UV treatment, p53R2 was activated and bound to hRRM1 to form RR holoenzyme to synthesize dNTP induced by UV, and then damaged DNA was impaired in the p53-mutant cell line PC3 [29]. DNMT/DNA adduct formation was a prerequisite for the activation of p53R2, and the p53R2 expression was induced by nucleoside-based DNMT inhibitors which could form DNA adducts [30]. It would take time to react to DNA damage after p53R2 induction, because p53R2 Ser72 phosphorylation by ataxia telangiectasia mutated (ATM) occurred within 30 min after genotoxic factors, and Ser72 phosphorylation by ATM was necessary for p53R2 stability and enduing resistance to DNA damage [13]. Besides, p53R2 dominant-negative or gain-of-function mutation was a major reason for mtDNA loss and mitochondrial disease [22]. Furthermore, a decreased p53R2 expression by siRNA significantly increased the cellular invasion potential in p53 mutant cell lines while the up-regulation of p53R2 could inhibit the tumor metastasis [31]. The different functions of p53R2 in different cancers indicated that p53R2 had two sides in tumorigenesis. Our study reveals that the protein level p53R2 is a novel factor for NPC patients with favorable prognosis. But the mechanism under the cell function and animal experiments need further exploration.

## Conclusions

In a conclusion, the examination of p53R2 expression, by IHC, could be used as an additional effective tool in identifying those NPC patients at favourable outcome. Our study may provide the evidence that p53R2 is a potential therapeutic target for NPC.

## Abbreviations

AUC: Area under the curve
IHC: Immunohistochemistry
NPC: Nasopharyngeal Carcinoma
RR: Ribonucleotide Reductase
